# 

**Published:** 1886-06

**Authors:** 


### INDEX.

### CONTRIBUTORS TO VOL. 1.

Abney, Dr. J. A .......... 3
Betz, Dr. Odo, Heilbronn, Germany .......... 529
Bennett, Dr. T. J .......... 12, 310
Beall, Dr. E. J .......... 413
Berry, Dr. D .......... 540
Baird, Dr. W. T .......... 67
Burt, Dr. W. J., .......... 73, 205
Bibb, Dr. R. H. L., Mexico .......... 537
Cochran, Irion & .......... 170
Christian, Dr. G. W., .......... 174
Cummings, Dr. J .......... 351
Collins, Dr. W. J .......... 318, 495
Carhart, Dr. J. W .......... 403
Doering, Prof. E. J .......... 220
Elston & Lanphear, Drs .......... ——
Francis, Dr. C. C .......... 159
Fisher, Dr. W. C .......... 395
Gregg, Dr. R. S .......... 545
Gwyn, Dr. C. L .......... 12, 261, 407
Ghent, Dr. H. C .......... 61
Greenwell, Dr. S. A .......... 85
Gibson, Dr. J. A .......... 136
Gregg, Dr. C. K., Mexico .......... 536
Hadra, Dr. B. E .......... 250
Irion & Cochran, Drs .......... 170
Kennedy, Dr. N. B .......... 266
Knox, Dr. R. T .......... 72
Keown, Dr. S. Leard .......... 172
Lanphear & Elston, Drs .......... 227
Lynch, Judge J. D .......... 441
Leake, Dr. H. K .......... 540
Lewis, Dr. J. M .......... 493

Morris, Dr. W. A .......................... 4, 538
Menger, Dr. R .......................... 546
McLaughlin, Dr. J. W .......................... 126
Marcy, Dr. H. O .......................... 299
Merryman, Dr .......................... 319
Moore, Dr. H. W .......................... 344
Osborn, Dr. T. C .......................... 109
Post, Dr. S. L .......................... 526
Paine, Dr. F. T .......................... 258, 315
Perkins, Dr. A. N .......................... 494
Penny, Dr. Wm .................. 228, 267, 324, 366, 410, 455
Swearingen, Dr. R. M .......................... 1
Smith, Dr. Q. C .......................... 971
Starley, Dr. S. F .......................... 59, 164
Schmidt, Dr. F. A .......................... 353
Turner, Dr. Peyton .......................... 357
Williams, Dr. R. G .......................... 67, 137
Wooten, Dr. T. D .......................... 117
White, Dr. R. W .......................... 319
Wolff, Dr. A. S .......................... 398
Walker, Dr. A. C .......................... 435

### CONTENTS TO VOL. 1.

ORIGINAL ARTICLES.

Alopecia, Premature Symptomtic .................... 3
A Long Cord .................... 137
An Extraordinary Case in Practice .................... 72
A Two Pound Child .................... 523
A Run of Bad Cases .................... 170
As Through a Smoked Glass—Darkly .................... 555
Ante-Partum Hæmorrhage .................... 350
Alcoholism .................... 403
Crypsorchis, A case of .................... 12
Carbolic Acid Injections in Carbuncle .................... 215
Carbuncle, Carbolic Acid Injection in .................... 215
Climatic Treatment of Disease .................... 299
Dengue in Austin, Remarks on .................... 205
Differentiation Between Chancre and Chancroid ...... 310
" " Diphtheria and Croup ...... 538–496
Electricity in Uterine Diseases .................... 59
" " Rheumatism and Neuralgia .................... 315

Eczma, How to Cure some cases of ........ 71
Ectrogenesis, a Case of ........ 73
Etiology of Tubercular Disease ........ 126
Emmett's Operation Followed by Pregnancy ........ 174
Enlarged Spleen Treated by Ergot Hypodermically ........ 407
Fistula in Ano, New Operation for ........ 136
Foreign Bodies in Rectum ........ 494
Gunshot Wound, a Rare Case of ........ 536
Gonorrheal Ophthalmia ........ 117
Hemorrhage from the Bladder ........ 61
" before delivery, A Case of ........ 220
" " " Letter from Germany ........ 529
Incision of Sphincter Vaginæ ........ 4
Lupus Exedens of the Face, A Case of ........ 109
Life and Character of Dr. Ashbel Smith ........ 441
Medical Etiquette ........ 159
Malarial Hæmaturia ........ 172
Malarial Irritation of Bladder ........ 540
Midwife's Diagnosis, A ........ 318
Milk in Fever ........ 319
Premature Symptomatic Alopecia ........ 3
Proper Mission of the Obstetric Forceps ........ 164
Peri-Uterine Cellulitis with Sequellæ ........ 261
Poisoning by Coal Gas, [2 Comanche Indians] ........ 344
Prize Essay ........ 555
Puerperal Eclampsia ........ 403
Placenta-Prævia Complicated with Hydatiforms Cysts, A Case of ........ 537
Revelations by Electricity ........ 258
Rupture of the Bladder, A Case of ........ 533
Review of Antiseptic Surgery ........ 485
Roundup, The ........ 554
Smith, Dr. Ashbel, Life and Character of ........ 441
Spinal Abscess, Remarks on ........ 250
Surgical Fracture of Femur ........ 1
Sympathetic Vomiting in Pregnancy ........ 67
Theory and Treatment of Diphtheria [Dr. Otto Ringk's] 353
Typhoid Fever, A Case with Complications ........ 13
Therapeutic Indications for Jaborandi ........ 9
Uræmia, Complicating Labor, A Case of ........ 359
Vaginal Hysterotomy ........ 495
Young M. D's. Soliloquy ........ 319

CULLINGS FROM CONTEMPORARIES.

Abstract from Foreign Exchanges ........ 320—363
All Wrong ........ 24
Abortion of Typhoid Fever ........ 178

Acute Coryza, A Parasitic Disease .................... 264
Cure of Aneurism by Insertion of Wire .... .......... 21
Curability of Consumption, The .................. .. 26
Cæsarian Section, after Death of Mother .............. 266
Dielectrolysis .... .............................. 225
Dr. Ferran's Experiment, [inoculation] ............... 77
Double Uterus, A Case of ......................... 22
Episiotomy ...................................... .. 149
Fœcal Abscess in Popliteal Space ..................... 361
How to build up a Medical Society .................. 264
Inoculation Gone Mad ............................... 409
Intelligent care of Dumb Brutes ...................... 222
Induced Abortion to relieve vomiting of Pregnancy.... 74
Intra-Peritoneal Adhesions .......................... 18
Laparotomy for Gunshot Wound of Intestines ........ 148
Latest Surgical Triumph, The......................... 27
Lupsus cured by Arsenic Internally .................. 21
Medical Iconoclast, A .............................. 362
Novel Methods of Entering the Bladder ............... 409
New Hæmostatic, A .................................. 226
Novel Expedient, A ................................. 91
Operative Treatment for Acute Intestinal Obstruction . 176
Prolonged Anæthesia from Cocaine .................. 174
Rupture of Perineum ................................ 15
Race of Life, The .................................. 80
"Sic Transit," Another Bubble Burst ................. 79
Traumatic Tetanus .................................. 149
Two New Local Anæsthetics ......................... 179
Unpleasant Accomplishment, An ...................... 81
Vaccination a Remedy for Whooping Cough ......... 224
Wholesome, but Unpalatable Facts ................... 264
Worth Knowing ...................................... 150

CORRESPONDENCE.

Letter from Dr. J. W. McLaughlin...............182, 28
" " " R. M. Swearingen.................... 83
" " " S. A. Greenwell...................... 85
" " " T. C. Osborn......................86, 180
" " Drs. Elston & Lamphear.................. 227
" " " Wm. Penny, "Our New York Letter"
.................228, 267, 324. 366, 410, 455
" " " N. B. Kennedy...................... 266
" " " H. K. Leake ........................ 322
" " " E. J. Beall, Von Brun's Bandage.... 413
" " " C. F. Paine.......................... 413
" " " R. S. Gregg, Lusus Naturæ........... 545
" " " R. Menger .......................... 546

EDITORIAL.

A Meaningless Act and What Came of it.............. 42
A Parallel..................... .......... 335
Another Stultification ............................ 553
Amende, The.................................... 338
Appointment, A Good............................ 337
A Journalistic Elsler.............................. 383
Advance in Surgery, A great....................... 432
Abbreviating Prescriptions.......................... 433
A Little Learning is a Dangerous Thing.............. 474
American Medical Association........................ 414
" " St. Louis meeting of the............... 520
American Medical Editors, Association of.........527, 419
Austin Medical Club....... ........................ 52
American Public Health Association.................. 327
Advertisers Notices, 54, 105, 156, 438, 200, 246, 295, 528, .........................340, 389, 482
Another Good Man Gone............................. 201
Bravo ........................................... 287
Blue's Elegy...................................... 288
Bilious Doctor The, to his Lady love................. 288
Bureau of Health.................................. 385
Beall, Dr. E. J.................................... 433
Benefaction, A.................................... 474
Baccillus Decalvans............................... 477
Billings, Dr. J. S.................................. 479
Biography of Contemporary Physicians of Texas .... 417
Burt, Dr. W. J..................................... 463
Bell County Medical Association..................... 464
Benefit, A ........................................ 202
Cholera, Precautions against........................ ——
Communism in Medicine............................ 92
Congratulation.................................... 428
Contemporary Physicians of Texas, Biography of...... 417
Convention Notes................................. 513
Christened ....................................... 53
Destruction of Shenanegan, The...................... 147
Dengue? Is it .................................... 233
Dengue Epidemic in Texas.......................... 190
Dengue, Micrococcus of............................. 238
Desseminating Truth and Doing Good, a la mode........ 194
Disgruntled Philanthropist, A........................ 199
Delinquents to Texas State Medical Association....... 242
Death of a Distinguished Physician of Texas........ 376
Death of Dr. Flint................................. 475
Death, The First in the P. M. B. A. of Texas............ 281

Death of Dr. W. H. Park ........ 290
Death of Dr. G. C. McGregor ........ 481
" of Dr· W. A. East ........ 339
Death of Dr. Calloway ........ 339
Diagnostic Sign, A New and Valuable ........ 284
Dr. Ashbel Smith, Death of ........ 376
" " " Life and Character of ........ 441
Disgraceful Act, A ........ 385
Dallas Meeting, of the T. S. M. A, The ........ 515
" County Medical Society, Action of ........ 89
Dangerous ........ 525
Delinquents, Notice to ........ 526
Death Benefit, Dr. W. H. Park ........ 325
Delegates, No Reduction in Fare to ........ 466
Defferentiation in Croup and Diphtheria ........ 496
Diphtheria and Croup, Differentition in ........ 496
Editors, Association of American Medical ........ 527
Et Tu Brute? ........ 96
Expedient, A Novel ........ 91
Ellis County Medical Society ........ 52
Errors in Transaction Texas Medical Association ........ 186
East Line Medical Society ........ 270
Essay, Prize for best ........ 420
Emulsion, An ........ 245
Florida Medical and Surgical Journal ........ 384
Flint, Dr. A. Death of ........ 475
Flint, Dr. A. and his Address ........ 549
False Hopes ........ 104
Good Showing, A ........ 443
Gush ........ 240
Gaillard's Medical Journal ........ 287
Grub to Catch Gudgeons, A ........ 480
Galveston Medical Club ........ 496, 373
Give us Your Names Gentlemen ........ 104
Heresy, False Doctrine and Schism ........ 33
Hold up Our Hands ........ 42
Homœopathic Strike, A ........ 553
Homœopaths, Why Physicians Cannot Consult With ........ 275
How Does Medical Journalism Aid the Profession ........ 423
Homœopathic Grammar ........ 429
How Medical Journalism has Aided the Profession in Texas ........ 469
Hymeneal 202, 295
Important Texas Work, An 47
International Medical Congress, The 9th 196, 338
Important Announcemeut 244
Journalistic Elsler, A 383

Knight, The Nameless .................................. 429
Lordly Fraud, A .................................. 378
Life and Character of Dr. Ashbel Smith .............. 441
Launched, (Salutatory .................................. 30
Mephistopheles, The N. Y. Journal and .............. 99
More Piracy .................................. 280
Married, Bennett, Johnson .................................. 339
" Cook, Radford, Baker .................................. 434
Maxwell, Dr. F. A. .................................. 339
Much Needed Work, A .................................. 469
Merited Compliment, A .................................. 473
Memorial .................................. 47
Mikado Medicus .................................. 526
Medical Society, Ellis Co .................................. 52
" " Austin Medical Club .................................. 52
" " Comanche County .................................. 53
" " Dallas Co. Action on I. M. Congress ... 89
" " Travis Co .................................. 151
" " East Line .................................. 270
" " Williamson County ..............271—36[illegible]
" " Milam " .................................. 371
" " Galveston Medical Club ..........496—373
" " Bell County .................................. 464
" " Cass County .................................. 466
Male Uterus, That .................................. 103
Melange ........................53, 103, 151, 202, 245—294
Miscellany .................................. 151
New Louisiana Quarantine .................................. 48
News from The Committee Meeting..........[Supplement]
Next .................................. 479
Our Turn .................................. 245
Overtures to Peace .................................. 285
Our Agents .................................. 288
Our Say on the Subject .................................. 380
On the Wrong Track .................................. 479
Principle vs. Policy .................................. 138
Plan Suggested to Regulate the Practice, A .......... 144
Professional Sentiment Evidenced by the Medical Press 197
Piracy and Obstinacy .................................. 338
Paine, Dr. F. T. .................................. 433
President of the 9th I. Med. Congress, The ........... 476
Physicians' Mutual Benefit Assn. of Tex., The 419, 91—187
151, 325
Physicians of Texas, Biography of Contemporary...... 417
Prize Essays .................................. 420
Programme for 18th Annual Convention T. S. M. A... 465
Precautions against Cholera .................................. 53

Quarantine on the Rio Grande .......................... 146
" in Texas .................................. 477
" , The New Louisiana ....... ............ 48
Rationale of the Action of Cocaine ..... ................ 37
Rauch, Dr. John H., of Ills ............................ 336
Regulating the Practice .............................. 337
Reminiscences of Canning ............................ 480
Salutatory, "Launched" ............................. 30
Salve for a Sore Head ................................ 40
Stultification, Another .............................. 553
Speculum, A New .................................... 200
Splenetic, Let the Galled Jade Wince ................ 551
Sauce for The Goose .... .......... ............. 237
State Medical Association, Texas ................... 240
Small, but Select .................................... 243
Small-pox in Texas .................................. 333
Smith, Dr. Ashbel .................................473—376
Self Elect, The ...................................... 432
Such is Fame ........................................ 478
Song of The Dying Swan, The ........................ 524
Seed Sown by the Wayside ............................ 271
Sorry Birds .......................................... 245
Stones and Glass Houses ............................. 295
St. Louis Meeting of T. S. M. A ...................... 520
Two-Edged Sword, A ................................. 284
Tug of War, The ..................................... 421
Texas Carries off the Honors ........................ 430
"The Association Captured" and Led Astray .......... 431
Texas Surgery ....................................... 471
Texas State Medical Association ..................515—240
" " " " Dallas, Meeting of .... 515
" " " " Transactions of 188, 53—150 186, 270
" " " " Preparation for meeting of ........................................ 327
" " " " Next Meeting of ...... 373
" " " " 18th Annual Meeting of ............................................ 476
" " " " Concerning the, Letter from Secretary ................................... 463
" " " " Programme of 18th Annual Meeting of ................................. 465
" " " " Summary of Proceedings of ............................................ 511
" " " " Delinquents to ....... 242
Texas Quackery, Circular on ........................ 372
Todgers Redivivus ................................... 54

Transactions of Texas State Med. Assn....188, 150, 186—270
Vice-President Hendrick's Disease .................... 288
Witty.................................................... 201
Wooten, Dr. T. D ....................................... 476
Who Shall Teach The Teachers......................... 523
Who ?.................................................... 431
Wants to Know, You Know .............................. 282
Ye Festive Bolter........................................ 427
Yellow Fever in Galveston............................... 285

BIBLIOGRAPHY.......................... 101, 188, 244, 292—386
Anatomy, Practical Human, Weiss ...................... 436
Diseases of Children, A Practical Treatise on............ 292
" of The Skin, Hand Book of.................... 292
" , A Treatise on Nervous....................... 386
" of Nose and Throat, Lectures on, Sajous ..... 387
" of The Eye, Pomroy ............................ 436
Don Miff, The Story of, Dabney ......................... 527
Electricity in Medicine ................................. 437
General Therapeutics, Ziemssen's Hand Book of ...101—386
How to Use Listerine ................................... 435
Index Catalogue of Library of Surgeon-General ........ 293
Index, Kansas City Medical - - - - 435
Kansas City Medical Index - - - 435
Milk Analysis and Infant Feeding, Meigs - - 386
Microscope, The Use of, Friedlander - - - 388
Nervous Diseases, A Treatise on - - - 386
Practice of Medicine, A Compendium of, Hughes - 434
Prairie Queen, The - - - - - - 435
Science and Art of Midwifery, The, [Lusk] - - 294
Text Book of Hygiene, A, [Rohe] - - - 103
Therapeutics, Hand Book of General - - - 101—386
Youth's Companion, The - - - - - - 389
Ziemssen's Hand Book of Genl. Therapeutics, - - 101—386

NECROLOGICAL, - - - - - - - - - 290—481
Death of Dr· Park - - - - - - - - 290
Death of Dr. G. C. McGregor - - - - - - - 481

